# Selection on start codons in prokaryotes and potential compensatory nucleotide substitutions

**DOI:** 10.1038/s41598-017-12619-6

**Published:** 2017-09-29

**Authors:** Frida Belinky, Igor B. Rogozin, Eugene V. Koonin

**Affiliations:** National Center for Biotechnology Information, National Library of Medicine, National Institutes of Health, Bethesda, Maryland USA

## Abstract

Reconstruction of the evolution of start codons in 36 groups of closely related bacterial and archaeal genomes reveals purifying selection affecting AUG codons. The AUG starts are replaced by GUG and especially UUG significantly less frequently than expected under the neutral expectation derived from the frequencies of the respective nucleotide triplet substitutions in non-coding regions and in 4-fold degenerate sites. Thus, AUG is the optimal start codon that is actively maintained by purifying selection. However, purifying selection on start codons is significantly weaker than the selection on the same codons in coding sequences, although the switches between the codons result in conservative amino acid substitutions. The only exception is the AUG to UUG switch that is strongly selected against among start codons. Selection on start codons is most pronounced in evolutionarily conserved, highly expressed genes. Mutation of the start codon to a sub-optimal form (GUG or UUG) tends to be compensated by mutations in the Shine-Dalgarno sequence towards a stronger translation initiation signal. Together, all these findings indicate that in prokaryotes, translation start signals are subject to weak but significant selection for maximization of initiation rate and, consequently, protein production.

## Introduction

The standard genetic code table contains one start codon, AUG. Over the years, however, many studies have demonstrated that alternative start codons, such as GUG, could be utilized for translation initiation with non-negligible frequencies, i.e. up to 20% non-AUG starts^1,2^. Recently, it has been shown that, under specific conditions, many different start codons can be used for initiation of translation in *Escherichia coli* although most of these are employed only rarely, whereas AUG, followed by GUG and then by UUG, remain the principal start signals^3^. In accord with this ranking of the start codons, genes starting with AUG are, on average, expressed at significantly higher levels than genes that start with GUG, and the latter are expressed at higher levels than genes starting with UUG^3,4^. This is generally the case for genes in other bacteria as well^3^ although it has been shown that GC content affects the frequency of genes starting with GUG compared with AUG^2^. Furthermore, in some bacteria, such as *Bacillus*, UUG is more prevalent and leads to higher levels of protein production than GUG^5^.

All start codons are apparently recognized by the dedicated initiator N-Formyl methionyl-tRNA with a CAU anticodon^6–8^. Recognition of the start codons and discrimination between the initiator and elongation tRNAs depends on the translation initiation factor IF3 that helps to position the correct start codon in the ribosomal P-site before binding the aminoacyl-tRNA^9,10^. In prokaryotes, the start codon is one of the major translation initiation determinants: replacement of AUG with an alternative start codon, such as GUG, typically leads to a several-fold drop in the translation efficiency^10–16^. In addition to the start codon, an important translation initiation signal is the Shine-Dalgarno (SD) sequence, also known as the ribosome-binding site, which base pairs with a complementary sequence near the 3′-terminus of the 16 S rRNA and increases the efficiency of initiation^17^. A correlation between the type of the start codon and the presence of the SD sequence has been reported: genes with an AUG start are more likely to possess an SD sequence than genes with alternative starts^18^. Translation of leaderless mRNAs that lack the SD sequence appears to require an AUG start codon, and this requirement does not depend on the anticodon complementarity to the start codon because compensatory modification of the anticodon does not restore translation of leaderless mRNAs with alternative start codons^19^. Furthermore, it has been shown that in leaderless mRNAs, the ribosomal 30 S subunit binds directly to the 5′-terminal AUG in the absence of the initiator tRNA^19^, and the proximity of the AUG to the 5′-terminus is an important determinant of initiation efficiency^20^. Thus, the start codon can be expected to be particularly important for initiation of translation in organisms that lack the SD sequence and have many leaderless transcripts, such as Cyanobacteria and some Archaea^21–24^. In addition to the start codon and the SD sequences, other sequences in the vicinity of the translation start site can affect the efficiency of initiation, in particular, several nucleotides immediately downstream of the start codon; moreover, compensatory relationships between such signals and the SD have been demonstrated^25–27^. Collectively, these findings indicate that the start codon in itself is a key translation initiation signal that is specifically recognized by the initiation machinery, in particular the ribosomal small subunit, in a tRNA-independent manner, and interacts with other initiation signals.

In an attempt to identify the factors that affect evolution of translation starts in prokaryotic genomes, we calculated frequencies of switches between start codons in groups of closely related bacterial and archaeal species. Comparison of these frequencies to those of the respective base triplet switches in non-coding sequences and the switches between the corresponding codons in coding sequence provides evidence of purifying selection on start codons that is, however, weaker than selection affecting the respective non-start codons. Additionally, we identified significant associations between start codon switches and substitutions in the upstream SD sequence and in positions −1 to −3.

## Methods

### Analysis of start codon switches

Genomic data for bacteria and archaea were obtained from an updated version of the ATGC (Alignable Tight Genome Clusters) database^28^. To reconstruct mutations in protein-coding and non-coding DNA by the parsimony principle, we used triplets of closely related species as previously described^29^. We analyzed 36 of the previously described 37 triples of genomes^29^ because in the species *Francisella noatunensis orientalis* all genes had an AUG start codon, and about 100 genes present in the other species in the same ATGC group (ATGC138) were missing, most likely, due to annotation errors. Therefore, the ATGC138 triplet was removed from the current analysis. Accurate analysis of start codon evolution critically depends on correct annotation of the start positions, which is known to be error-prone^4^. Therefore, elimination of unreliable start annotations is crucial in such analyses. To reduce the impact of erroneous start annotations, we only used genes, for which the annotated start positions from the three compared genomes aligned precisely in all genomes of the respective ATGC. We further verified that removal from the analysis of genes with a conserved putative start codon within 60 nucleotides upstream or 60 nucleotides downstream of the annotated start did not significantly alter the calculated switch frequencies (Table S1). Alignments of all sequences in each ATGC COG (Cluster of Orthologous Genes) were constructed using the MAFFT software with the –linsi algorithm^30^. Only cases where the outgroup species start codon (or other control state) was identical to that of one of the ingroup species was considered in the analysis to limit the calculated switches to only one of the branches leading the ingroup species from their common ancestor (Fig. S1A,B). The standard error for the frequencies is calculated as $\sqrt{{pq}/n}$.

Potential limitations of this analysis are the assumptions of parsimony. In particular, parsimony does not account for the possibility of multiple substitutions in the same position along the same branch, because of which some switch frequencies could be under- or over-estimated. However, the effect of this assumption is likely to be minimal in our setting because the genomes used to calculate the switch frequencies are closely related, which decreases the probability of multiple substitutions in the same position. Using FastML^31^, we verified that Maximum Likelihood reconstruction strongly agrees with the ancestral state reconstructed under parsimony (Table S2).

### Evolutionary rate estimation

Taking into account ATGCs with at least 12 genomes and at least 1000 genes, the median *dN/dS* (ratio of the estimated rates of non-synonymous and synonymous substitutions) value of all genome pairs in a group was used as the proxy for the selection strength at the protein level^32^. The *dN/dS* values were estimated using the Codeml program^33^. In each ATGC, all genes were divided into 3 sets based on the start codon (AUG, GUG or UUG). The significance of the differences between the *dN/dS* values for genes starting with different start codons was determined using Wilcoxon rank sum test. The significance of the differences between the counts of genes with *dN/dS* above or below the overall *dN/dS* median of each ATGC was determined using Fisher’s exact test.

### Protein abundance analysis

Integrated protein abundance levels for *E. coli* K12 MG1655 were downloaded from PaxDb^34^. Each protein id was mapped to its RefSeq geneID, for which the start codon was extracted from the whole genome sequence NC_000913.3. Taking into account only genes with protein abundance above the median (3.95 ppm), the protein abundances for genes starting with AUG, GUG and UUG were compared using Wilcoxon’s rank sum test.

### Cumulative substitution scores around start codons

The positions from −20 to +9 were extracted from each genome of all ATGC COGs and subjected to pairwise comparison per position. To reduce impact of erroneous start annotations, only genes with starts aligning precisely to the majority of the genomes within a given ATGC. The majority rule was used as an approximation for the correct start codon position, in order to keep as many informative data points as possible, for comparison of strictly homologous start codons. This filtering procedure was designed slightly differently from that applied in the triplet design used for the evolutionary rate analysis described above, in order to incorporate more data. When the base in a certain position was identical in both genomes, the position was assigned a value of 0, and a value of 1 was assigned when the bases were different. For each COG, the sum of all pairwise comparisons was assigned a maximum value of 1 for a given position under the conservative assumption that a substitution in each position in each ATGC COG occurred only once. This is an underestimation of the actual number of substitutions which is slightly greater; positions with more than 2 possible nucleotides comprise about 8% of all variable positions in the analyzed data. All COGs, for which the maximum number of pairwise substitutions in the 29 base window was smaller or equal to 15, were further considered, in order to reduce the chance of comparing non-homologous sequences. Non-homologous sequences could arise due to indels, HGT, or gene duplication. Random sequences of 29 bases with two positions being constant (the last 2 position of the start codon) are equivalent to 27 positions with 4 possible bases for each position, equivalent to binomial distribution with N = 27 and P = 0.25, for which the average number of matches is NP = 6.75 and therefore, the average number of mismatches in 29 positions with two constant ones is 20.25 and the standard deviation is (NP(1-P))^5^ = 2.25. Thus, 15 mismatches is more than 2 standard deviations away from the mean and therefore most likely result from substitutions in homologous sequences. We further verified that applying a more stringent threshold of no more than 10 substitutions does not have a major effect on the results (Fig. S2). For any given feature (e.g., a specific start codon or a specific phylum), all substitutions in the 29 base windows from all qualifying COGs with that feature were summed up. Statistical significance of the differences between features (i.e., different start codons or different SD strength) was calculated using a chi-square test, which was performed separately for the upstream region (−20 to −1 with 95 df) and the coding region ( + 4 to + 9 with 30 df).

### Shine-Dalgarno sequence strength

We defined any gene that contains AGGA, GGAG, or GAGG in positions −20 to −5 as having a strong SD sequence, any gene with AG, GG, or GA in positions −20 to −5 as having a weak SD, and all the rest of the genes as having no SD. Although SD motifs could be different in diverse bacterial species due to divergence of the anti-SD sequence at the 3′ end of 16 S rRNA, this definition is expected to capture all SD sequences because the core CCUC of the anti-Shine-Dalgarno that is present in all prokaryotes^18^.

### Detection of coupled substitutions

To detect potential coupled substitutions, we first identified all cases in which exactly two substitutions were present in the 29 base window from −20 to +9. We then sought to detect those pairs of substitutions that are significantly more frequent than other pairs. Specifically, we were interested in substitution pairs where one of the substitutions is in the start codon (i.e., position +1) to pinpoint potential cases of compensatory substitutions for start codon switches. To this end, the substitution pairs were divided into 3 groups (Fig. S3): (i) a substitution in position +1 and another substitution in position −1, −2 or −3, (ii) a substitution in position +1 and another substitution in position −7, −8, −9 or −10, (iii) a substitution in position +1 and another substitution anywhere else (i.e., position −4, −5, −6, −11 to −20, or + 4 to +9). The control groups in Fisher’s exact test for these substitution groups were: (i) a substitution in any position but +1 and another substitution in positions −1, −2 or −3, (ii) a substitution in any position but + 1 and another substitution in positions −7, −8, −9 or −10, (iii) a substitution in any position but +1 and another substitution anywhere else (i.e., position −4, −5, −6, −11 to −20, or + 4 to +9).

To identify potential compensatory evolution involving SD sequences and start codons, we identified, within the 20 bases upstream of the start codon, ‘switches’ in SD, i.e. cases when a strong SD (AGGA, GGAG or GAGG) switched to a weak SD (AG, GG, or GA) or vice versa. We also documented all non-switched cases of strong or weak SD motifs in combination with any of the start-codons or start codon switches.

## Results

### Start codon switches

**Figure 1:**
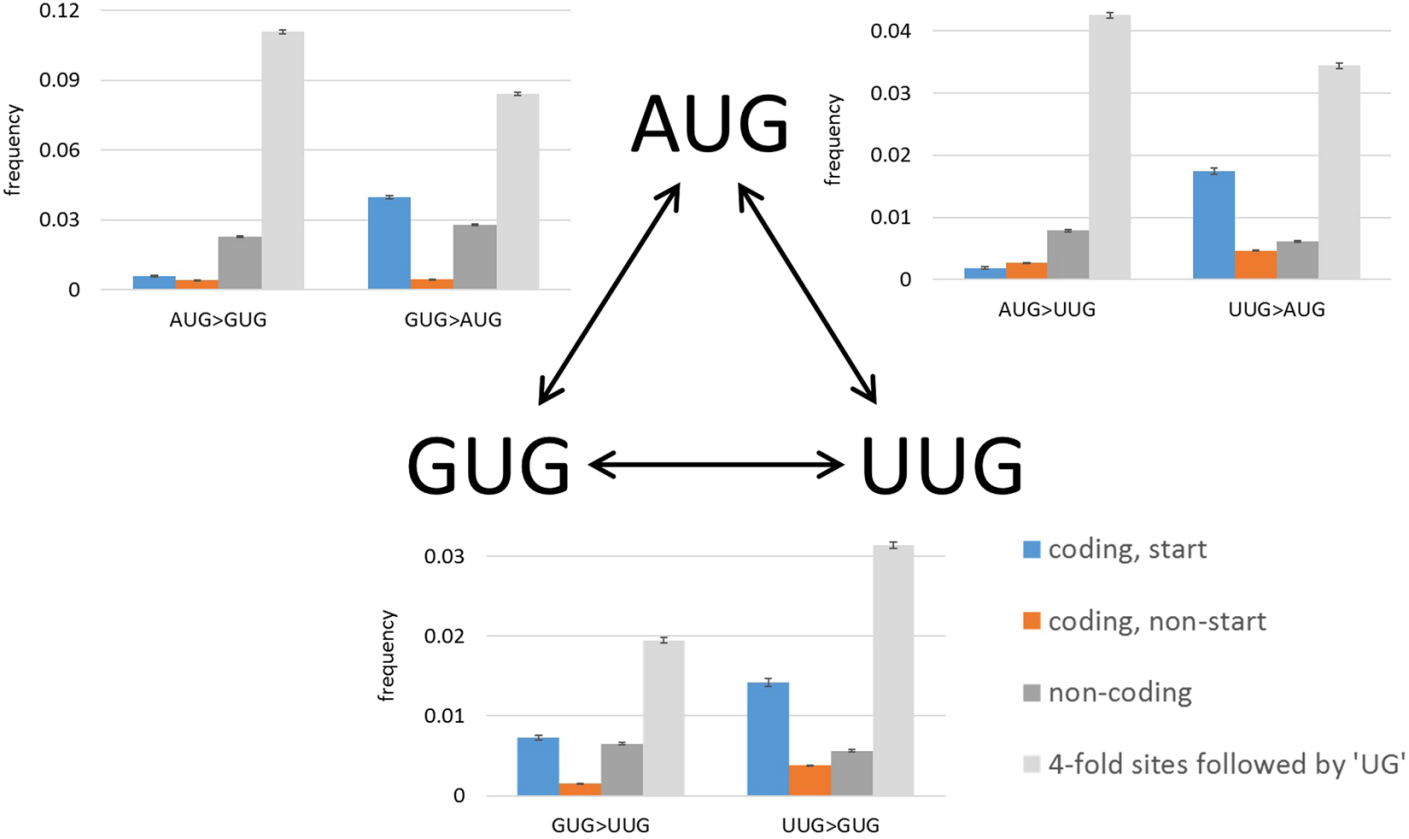
Selection on start codons switches. The 3 panels compare the frequencies of each of the switches between the 3 start codons (blue), the respective codons in non-start positions of coding sequences (orange), the corresponding nucleotide triplets in non-coding sequences (dark grey), and the corresponding 4-fold degenerate sites that are followed by ‘UG’ (light grey). Error bars indicate standard errors of the frequencies.

Following the previously implemented strategy^29^, we used 36 triplets of closely related genomes with unambiguous phylogeny to reconstruct start codon substitutions (Fig. 1, Table 1). Of the 61,358 reconstructed ancestral AUG starts, 363 switched to GUG (0.0059), 113 switched to UUG (0.0018), 2 switched to CUG (3.2 × 10^−5^), and 1 switched to AUU (1.6 × 10^−5^). Of the 5,080 reconstructed ancestral GUG starts, 202 switched to AUG (0.0398), 37 switched to UUG (0.0073), 1 switched to CUG (2 × 10^−4^), and 1 switched to AUU (2 × 10^−4^). Of the 2,468 ancestral UUG starts, 43 switched to AUG (0.0174), 35 switched to GUG (0.0142), and 1 switched to CUG (4 × 10^−4^). The frequency of start codon substitutions to an AUG start codon was significantly higher than the frequency of substitutions from an AUG (p = 4.6 × 10^−79^ for GUG, p = 1.7 × 10^−24^ for UUG). Stat codon switches from UUG to GUG also were significantly more frequent than reverse switches from GUG to UUG, albeit with a lesser significance, due to a much smaller sample (p = 0.005). We further demonstrated that the difference in the frequencies of switch to an AUG and from an AUG start codon was significant in each of the individual prokaryotic phyla except for γ-proteobacteria, where the difference between the frequencies of the AUG to UUG and UUG to AUG switches was found not to be significant (Table 2). Apart from this consistency, comparison of the frequencies of start codons and their switches in different phyla shows some notable anomalies (Table 2). For example, in Actinobacteria, about 30% of the ancestral start codons are GUG, whereas Clostridia show substantially greater rate of the GUG to AUG switches, with 10% of the GUG codons changed to AUG.

**Table 1:** Start codon switch counts and frequencies in 36 triples of prokaryotic genomes compared to the switches of the same nucleotide triplets in non-start coding regions and non-coding regions.

Switch	Start codon switch Count	Start codon switch frequency	Non-coding switch Count	Non-coding switch frequency	4-fold sites switch count	4-fold sites switch frequency	Coding non-start switch Count	Coding non-start switch frequency
AUG > GUG	363	0.0059	2,273	0.0228	1,564	0.1107	2,623	0.004
AUG > UUG	113	0.0018	783	0.0079	601	0.0425	1,790	0.0027
GUG > AUG	202	0.0398	2,370	0.0279	2,807	0.0842	3,343	0.0046
GUG > UUG	37	0.0073	553	0.0065	649	0.0195	1,106	0.0015
UUG > AUG	43	0.0174	722	0.0061	628	0.0344	948	0.0047
UUG > GUG	35	0.0142	659	0.0056	573	0.0314	771	0.0038

**Table 2:** Start codon switch frequencies in well-sampled prokaryotic phyla.

	AUG > UUGUUG > AUG			AUG > GUGGUG > AUG			GUG > UUGUUG > GUG			Ancestral codon counts		
	#	Freq.	p Fisher	#	Freq.	p Fisher	#	Freq.	p Fisher	AUG	GUG	UUG
α-proteobacteria	244	0.0030.012	0.007	6234	0.0050.043	1.2e–16	66	0.0070.019	0.12	11440	789	323
β-proteobacteria	134	0.0110.021	1.6e–4	6215	0.0050.029	1.3e-6	43	0.0080.016	0.4	11347	520	191
γ-proteobacteria	191	0.0010.004	0.25	6337	0.0040.030	7.2e–19	52	0.0040.008	0.35	17733	1219	258
δ/ε-proteobacteria	13	3e-40.022	3e-4	2414	0.0080.019	0.01	02	00.014	0.03	3178	735	139
Bacilii	3716	0.0040.020	1.5e-6	6129	0.0060.04	1.3e-12	713	0.0100.016	0.37	9861	725	820
Clostridia	97	0.0040.022	0.002	4324	0.0190.103	9.6e-9	74	0.0300.013	0.21	2224	233	315
Actinobacteria	42	0.0020.051	0.0065	2737	0.0150.062	2.7e–8	11	0.0020.026	0.12	1850	598	39
Methanococci	34	0.0020.040	0.001	34	0.0020.080	9.1e–5	01	00.010	1	1219	50	101

### Comparison of start codon switches to corresponding triplet switches in non-coding regions

In non-coding regions, we identified 99,677 ancestral AUG triplets, of which 2,273 switched to GUG (0.0228) and 783 switched to UUG (0.0079). Of the 85,010 ancestral GUG triplets, 2,370 switched to AUG (0.0279) and 553 switched to UUG (0.0065). Of the 117,423 ancestral UUG triplets, 659 switched to GUG (0.0056) and 722 switched to AUG (0.0061). Comparison of the switch frequencies in non-coding regions to switches in start codons shows that switches to an AUG start codon are significantly more frequent than switches to AUG in non-coding regions (p = 8.0 × 10^−9^ for UUG to AUG switches, and p = 5.6 × 10^−6^ for GUG to AUG switches). Conversely, switches from an AUG start codon were significantly less frequent than equivalent switches in non-coding regions (p = 1.3 × 10^−166^ for AUG to GUG switches, and p = 1.9 × 10^−64^ for AUG to UUG switches). The frequencies of switches from GUG to UUG were not significantly different between start codons and non-coding regions (p = 0.474), whereas switches from UUG to GUG were significantly more frequent in start codons vs. non-coding regions (p = 2.6 × 10^−6^).

### Comparison of start codon switches to corresponding triplet switches in 4-fold degenerate sites

Although non-coding DNA is generally less conserved than protein-coding genes, it is not free of purifying selection^29^. To further compare start codon switches to the same nucleotide substitutions in sequences that are minimally affected by selection and better reflect possible mutation biases, we compared start codon switches to corresponding switches in 4-fold degenerate sites (i.e., synonymous codons within protein coding genes that can contain any of the 4 bases in the third position). In 4-fold degenerate sites that are followed by ‘UG’, we identified 14,127 ancestral AUG triplets, of which 1,564 switched to GUG (0.1107), and 601 switched to UUG (0.0425). Of the 33,335 ancestral GUG triplets, 2,807 switched to AUG (0.0842) and 649 switched to UUG (0.0195). Of the 18,250 ancestral UUG, 628 switched to AUG (0.0344), and 573 switched to GUG (0.0314). All start codon switches were significantly less frequent than the corresponding changes in 4-fold degenerate sites (p = 3.57 × 10^−312^ for AUG > UUG, p = 9.88 × 10^−324^ for AUG > GUG, p = 1.12 × 10^−28^ for GUG > AUG, p = 3.28 × 10^−11^ for GUG > UUG, p = 5.29 × 10^−7^ for UUG > GUG, and p = 3.32 × 10^−6^ for UUG > AUG). To test whether unequal frequencies of nucleotide triplets in start sites, coding and non-coding regions might affect the calculated switch frequencies, we sampled positions from each control group to match the AUG, GUG and UUG start codon frequencies and recalculated the switch frequencies from 1000 replicates. We observed that the mean switch frequencies calculated using this sampling procedure were (nearly) the same as the original frequencies (Table S3).

### Selection on start codons is weaker than selection on the same codons in coding sequences

We further compared the frequencies of start codon switches to the frequencies of the same codon switches in coding regions, in which case these substitutions result in amino-acid substitutions. We identified 660,975 ancestral AUG triplets in coding sequences, of which 2,623 switched to GUG (0.004) and 1,790 switched to UUG (0.0027). Of the 733,352 ancestral GUG triplets, 1,106 switched to UUG (0.0015) and 3,343 switched to AUG (0.0046). Of the 203,189 ancestral UUG triplets, 771 switched to GUG (0.0038) and 948 switched to AUG (0.0047). Switches between AUG, GUG and UUG codons in coding regions are significantly less frequent than in non-coding regions (p = 6.4 × 10^−323^ for AUG > GUG, p = 2.4 × 10^−323^ for GUG > AUG, p = 3.8 × 10^−115^ for AUG > UUG, p = 3.2 × 10^−8^ for UUG > AUG, p = 8.0 × 10^−140^ for GUG > UUG, and p = 3.2 × 10^−13^ for UUG > GUG), as could be expected under the assumption that many amino acid residues in proteins are subject to purifying selection. Comparison of the frequencies of coding non-start switches to the frequencies of start codon switches shows that the switch from AUG to UUG occurred significantly less frequently in start codons than in coding regions (p = 3.1 × 10^−5^), whereas all other switches were significantly less frequent in coding regions compared to the start codon switches (p = 1.4 × 10^−11^ for AUG to GUG, p = 3.5 × 10^−111^ for GUG to AUG, p = 2.0 × 10^−12^ for UUG to AUG, p = 3.6 × 10^−14^ for GUG to UUG, and p = 2.0 × 10^−10^ for UUG to GUG). Taken together with the results in the previous section, these findings indicate that start codons are subject to purifying selection (preserving AUG) that, however, is significantly weaker than the selection on the corresponding amino acid substitutions, except for AUG to UUG changes, which are subject to purifying selection that is significantly stronger compared to Met to Val changes in coding regions.

### Correlation between selection on start codons and selection on protein sequences

**Figure 2:**
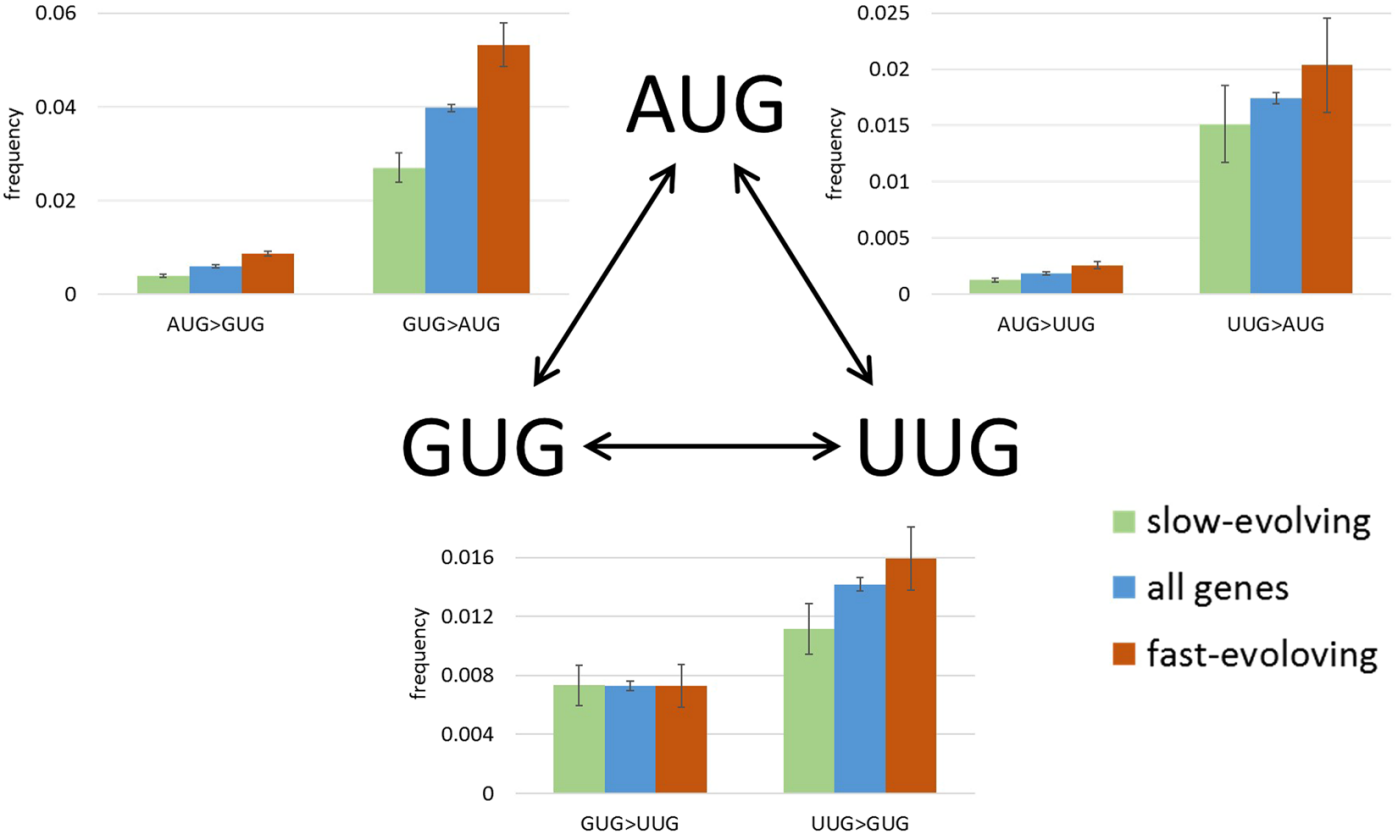
Selection on start codon switches in slow-evolving and fast-evolving genes. Error bars indicate standard errors of the frequencies.

We divided all genes into slow evolving and fast evolving groups, where slow evolving are genes with *dN/dS* lower than the median of the genomic distribution, and fast-evolving genes are those with *dN/dS* higher than the median for each ATGC triplet (Fig. 2, Table 3). Slow-evolving genes contained 32,216 ancestral AUG start codons, of which 126 switched to GUG (0.0039) and 40 switched to UUG (0.0012). Of the 2,592 ancestral GUG start codons in slow-evolving genes, 70 switched to AUG (0.027) and 19 switched to UUG (0.0073). Of the 1,255 ancestral UUG start codons in slow-evolving genes, 14 switched to GUG (0.011) and 19 switched to AUG (0.015). In fast-evolving genes, we identified 26,829 ancestral AUG start codons, of which 230 switched to GUG (0.0086) and 69 switched to UUG (0.0026). Of the 2,330 ancestral GUG start codons in fast evolving genes, 124 switched to AUG (0.0532) and 17 switched to UUG (0.0073). Of the 1,129 ancestral UUG start codons in fast-evolving genes, 23 switched to AUG (0.0204) and 18 switched to GUG (0.0159). The frequency of switches that change an AUG to an alternative start codon in fast-evolving genes was significantly higher than in slow- evolving genes (p = 4.7 × 10^−13^ for AUG to GUG and p = 2.2 × 10^−4^ for AUG to UUG), as was the frequency of changing GUG to AUG (p = 6.7 × 10^−6^). Other switch frequencies were not significantly different between slow and fast evolving genes (p = 1 for GUG to UUG, p = 0.37 for UUG to GUG, and p = 0.35 for UUG to AUG). Thus, the evolution of start codons in fast-evolving genes is also faster than in slow-evolving ones. This observation implies that, in fast-evolving genes, purifying selection against switching from AUG to alternative starts is weaker than in slow-evolving genes.

**Table 3:** Start codon switch counts and frequencies in slow vs. fast evolving genes.

Start codon switches	All genes switch count	All genes switch frequency	Slow evolving genes switch count	Slow evolving genes switch frequency	Fast evolving genes switch count	Fast evolving genes switch frequency
AUG > GUG	363	0.0059	126	0.0039	230	0.0086
AUG > UUG	113	0.0018	40	0.0012	69	0.0026
GUG > AUG	202	0.0398	70	0.027	124	0.0532
GUG > UUG	37	0.0073	19	0.0073	17	0.0073
UUG > AUG	43	0.0174	19	0.015	23	0.0204
UUG > GUG	35	0.0142	14	0.011	18	0.0159

### Evolutionary rates and protein abundance of genes with different start codons

**Figure 3:**
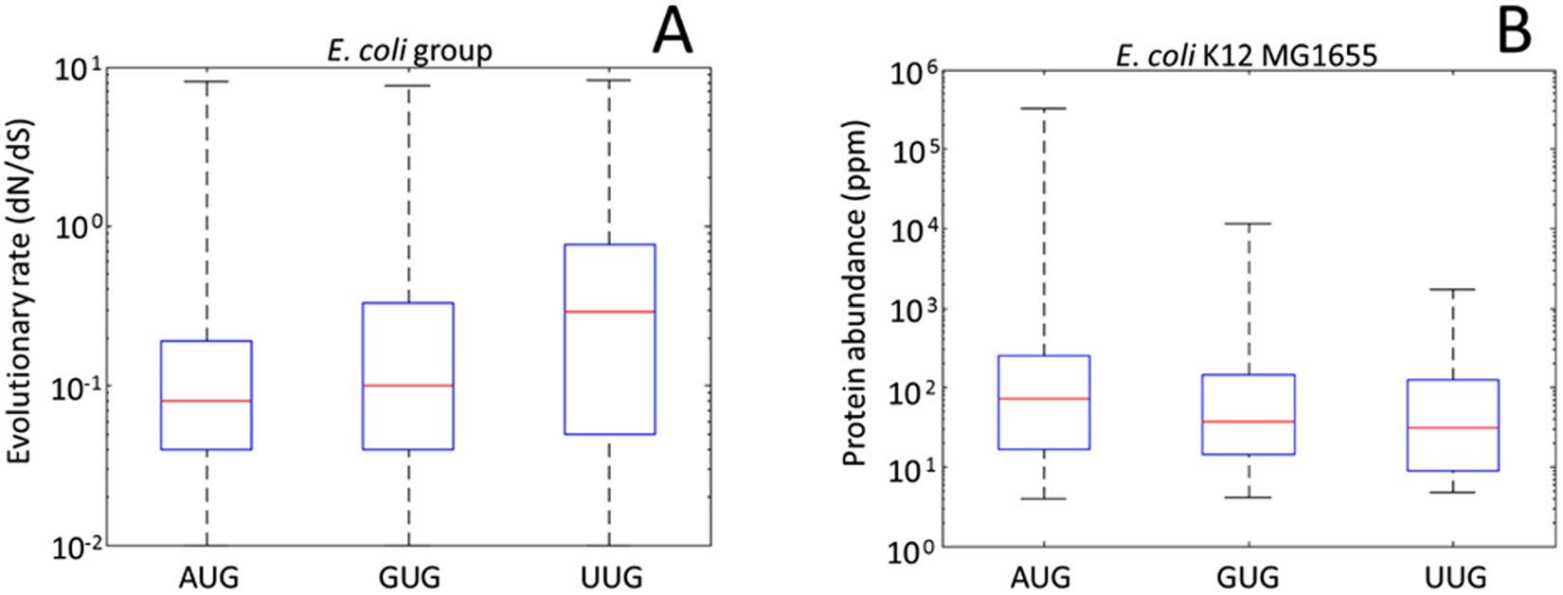
Comparison of the evolutionary rate (**A**) and protein abundance (**B**) in *Escherichia coli* genes starting with AUG, GUG or UUG codons. The lower bound of dN/dS values is zero. To enable presentation in log scale the lower bound was shifted to a finite value that was arbitrarily set to 0.01.

In ATGC001 (the group including *E. coli*), the evolutionary rate of genes starting with AUG is significantly lower than that of genes starting with GUG and UUG (Fig. 3A; p = 7.4 × 10^−20^ and p = 2.7 × 10^−5^, respectively), and the evolutionary rate of genes starting with GUG is significantly lower than that of genes starting with UUG (p = 8.8 × 10^−8^). A similar trend was observed in 14 other ATGCs of the 21 examined ATGCs with at least 12 genomes and at least 1000 genes (Fig. S4 and Table S4).

When comparing protein abundance based on start codon, analysis of all genes showed a borderline result (p = 0.011 for AUG vs. GUG and p = 0.755 for AUG vs. UUG, p = 0.376 for GUG vs. UUG). However, among genes with abundance greater than the median, proteins encoded by genes starting with AUG are, on average, more abundant than proteins encoded by genes starting with either GUG or UUG (Fig. 3B, p = 0.004 and p = 0.01, respectively); genes starting with GUG and UUG were not significantly different in terms of protein abundance, conceivably, due to the lower total numbers (p = 0.3).

### Coupling of start codons and start codon switches with the evolution of adjacent sequences

**Figure 4:**
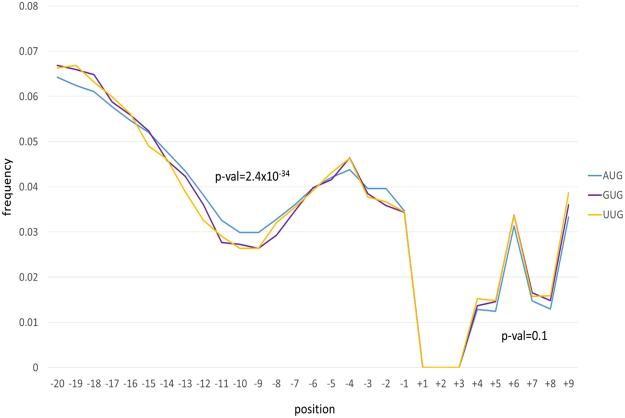
Cumulative substitution frequencies in 29 base pair windows.

In genes starting with AUG, GUG or UUG (genes starting with other codons such as CUG or AUU are few and were not included in this analysis), a consistent trend of cumulative substitutions was observed (Fig. 4), whereby the region between positions −13 and −7, i.e. the SD sequence that base-pairs with 16 S rRNA, accumulates fewer substitutions than other adjacent sequences. Notably, genes starting with GUG or UUG show significantly stronger conservation (fewer cumulative substitutions) in the SD region compared to genes starting with AUG (p = 2.4 × 10^−34^), suggestive of compensatory evolution for genes with either weak or strong Shine-Dalgarno motifs (Fig. S5). However, analysis of individual phyla shows that this is not a universal trend (Fig. S6). Thus, in α- proteobacteria, the difference in cumulative substitutions between genes starting with different start codons is not significant (Fig. S6A). In γ- proteobacteria, only genes starting with GUG have a more conserved SD area compared to genes starting with AUG (Fig. S6B), whereas genes starting with UUG have a more conserved −1 to −3 region. In Firmicutes (including Clostridia and Bacilii), both genes starting with UUG and GUG have a more conserved SD area compared to genes starting with AUG (Fig. S6C), whereas in Archaea, the difference in the SD conservation level was significant only for UUG vs AUG (Fig. S6D).

To identify possible concerted or compensatory substitutions, we analyzed all pairs of substitutions in the −20 to +9 region (Table 4). Of the 9,741 substitutions coupled with substitutions in positions −7 to −10 (the most conserved subsequence of the SD sequence), 80 start codon switches were identified (0.0082). Of the 15,529 substitutions coupled with substitutions in positions −1 to −3, we identified 118 start codon switches (0.0076), and among the 76,884 substitutions coupled with a substitution in any other positions, there were 405 start codon switches (0.0053). Start codon switches coupled with substitutions in positions −7 to −10 were significantly more frequent than start codon substitutions coupled with any other positions except for −1 to −3 (p = 5.2 × 10^−4^). Similarly, coupling with start codon substitutions was more common in positions −1 to −3 compared to all other positions except for −7 to −10 (p = 6.7 × 10^−4^). No significant difference was found between start codon substitutions coupled with substitutions in positions −7 to −10 and with positions −1 to −3 (p = 0.61). These observations suggest that substitutions in the Shine-Dalgarno sequence as well as positions −1 to −3 compensate for start codon switches.

**Table 4:** Coupled substitutions in start codons and neighboring sequences.

Paired with a single substitution at position	Start codon substitutions	All other substitution	Frequency	p-value of comparison to all other positions	p-value of comparison to −1 to −3	p-value of comparison to −7 to −10
−7 to −10	80	9741	0.0082	5.2 × 10^−4^	0.61	—
−1 to −3	118	15529	0.0076	6.7 × 10^−4^	—	0.61
All other positions	405	76884	0.0053	—	6.7 × 10^−4^	5.2 × 10^−4^

Among genes starting with AUG, 20,597 had a strong SD and 12,340 had a weak SD. Among genes starting with GUG, 1,631 had a strong SD and 818 had a weak SD. Among genes starting with UUG, 959 had a strong SD and 392 had a weak SD. Thus, in agreement with the results of the cumulative substitution analysis (Fig. 4, Fig. S2), genes starting with AUG are significantly less likely to have a strong Shine-Dalgarno compared to genes starting with either GUG (p = 5.5 × 10^−5^) or UUG (p = 1.6 × 10^−10^). These observations appear indicative of compensatory evolution between start codons and SD. Combinations of Shine-Dalgarno and start codon switches did not exhibit any statistically significant associations, and neither did combinations of Shine-Dalgarno switches and particular start codons, or combinations of start codon switches and particular Shine-Dalgarno strengths (Table S5).

## Discussion

Analysis of the evolutionary switches between start codons in groups of closely related prokaryotic genomes reveals purifying selection against substitutions that alter ancestral AUG starts. This pattern can be deduced from the significantly lower frequencies of switches from AUG start codons to GUG and UUG compared to the reverse switches (Fig. 1). Evolution of the start codons under purifying selection is supported by comparison to switches in non-coding regions and in 4-fold degenerate sites, which are largely symmetrical. These results could be expected simply from the fact that AUG is the predominant start codon in all prokaryotes but, to our knowledge, direct evidence of purifying selection on start codons has not been previously reported. It has been proposed that GUG and UUG, which are considered weaker start codons, are used to allow for specific regulation in combination with a weak Shine-Dalgarno sequence and a secondary structure masking it^35^. Although this might be the case for some genes, our findings show that, on the genome scale, selection for AUG, as the optimal start codon, and against non-AUG start codon is readily detectable. Furthermore, our findings are compatible with the possibility of positive selection driving the switches from GUG and UUG to AUG although we did not obtain direct evidence of this mode of evolution.

Comparison of the frequencies of start codon switches to the frequencies of the same codon switches in coding sequences shows that purifying selection against these substitutions is, on average, significantly stronger in coding sequences than it is in start codons (Fig. 1). Given that all start codons encode bulky hydrophobic amino acids, so that these codon switches lead to conservative amino acid substitutions, our results imply weak purifying selection on start codons.

In the current setting of codon switch analysis that included only triples of related genomes and in the absence of a maximum likelihood substitution model optimized for start codons, we could not directly demonstrate that start codons were in equilibrium. Nevertheless, comparison of all start codons in genome triples from all analyzed groups of bacteria and archaea showed no significant change in the frequencies of all 3 start codon from the outgroup to the ingroups (Table S6, p = 1). Thus, although based on the switch frequencies calculated from the ancestral start codon counts, one might have concluded that the frequency of AUG was decreasing, this is not the case. The cause of this discrepancy is that, in the current setting, the cases where the two ingroup genomes had identical start codons that were different from the outgroup were ignored (Fig. S1C). These cases include many switches to AUG in the internal branch leading to the ingroup species that were excluded from the switch analysis, in order to meet the parsimony assumptions. Taking these switches into account would add back many AUG starts that are lost in the short external branches.

The rates of start codon switches correlate with the rate of evolution (or selection strength) of the corresponding coding sequences. In fast-evolving genes, the frequencies of both the AUG to GUG switch and the reverse switch from GUG to AUG were significantly greater than in slow-evolving genes. The weaker conservation of AUG is fast-evolving genes is in line with the overall weak purifying selection. The increased rate of the GUG to AUG switches appears somewhat unexpected. This observation could be explained by purifying selection on a subset of the GUG starts that is stronger in slow-evolving than in fast-evolving genes. The positive correlation between the selection on AUG start codons and protein sequences is further demonstrated by the lower *dN/dS* of genes starting with AUG in most groups of prokaryotes. This connection between the start codon and the strength of selection affecting the respective protein-coding sequence is most likely related to the well-established anticorrelation between the level of gene expression (or protein abundance) and evolutionary rate of protein sequences^36^. Indeed, we observed that proteins encoded by genes starting with AUG, on average, are more abundant than those starting with other codons, which correlates with their slower evolution.

In some species, the GC content has been shown to affect the ratio of the AUG to GUG start codons^2^. In accord with these observations, we found that the frequency of switches from GUG to AUG was significantly higher in genome triples with low GC content than in those with high GC content (Fig. S7). However, the frequency of switches from AUG to GUG was universally low and not significantly different between these organisms, emphasizing the dominant role of purifying selection in the evolution of the start codons.

Notably, we detected apparent compensatory evolution of the start codons and additional translation initiation signals. Genes starting with GUG and UUG typically show a stronger conservation of the Shine-Dalgarno sequence compared to genes with an AUG start (Fig. 4). Although an early study has reported a positive correlation between the presence of an SD sequence in a gene and an AUG start codon^18^, our results suggest that the role of SD in translation initiation is greater in genes with non-AUG start codons (Fig. 4). However, that this trend differed among the analyzed groups of prokaryotes, implying existence of additional regulatory interactions. Because both the SD and the surrounding sequence are likely to evolve under purifying selection, the comparison of substitutions in the SD area to the substitutions in the surrounding sequences appears to be a better control than near neutral positions, such as 4-fold degenerate sites. Moreover, because we show that, in general, the SD area is more conserved (i.e. subject to stronger purifying selection) than the upstream and downstream positions, the elevated number of substitutions in the SD when coupled with start codon switches is unlikely to be observed under the neutral evolutionary regime. Accordingly, we interpret this coupling as either a response or a precondition to many start codon switches. Due to the small number of start codon switches combined with changes in the strength of the SD motif, we could not conclusively determine whether the direction of changes was synergistic or compensatory. However, the significant association of AUG with weak SD motifs as opposed to stronger SD motifs associated with non-AUG start codons suggest compensatory evolution of the translation start and SD.

In addition to the correlated evolution of the start codons and the SD sequence, we detected significant coupling between the start and positions −1 to −3 (Table 4). Substitutions in these positions have been shown previously to substantially affect protein expression^37^. Furthermore, position −1 has been specifically implicated in maintaining the stability of base-pairing with formyl-methionine tRNA^38,39^. In Cyanobacteria, a di-pyrimidine in positions −1 and −2 has been suggested to play a role in translation initiation^23,40,41^. Taking into account all these lines of evidence, our finding suggests that positions −1 to −3 represent yet another translation initiation signal in many prokaryotes.

Additional compensatory mechanisms affecting the efficiency of translation are likely to exist. For example, evidence has been presented that, similarly to start codons, stop codons are also subject to purifying selection that appears to remove UAG stops in bacteria^42^. Future work including direct calculation of stop codon switch frequencies should further elucidate this matter.

Taken together, the results presented here translate into a simple model of start codon evolution in prokaryotes. Under this model, AUG is the optimal start codon, and the detectable forms of selective pressure mostly act to maintain or create this optimal start. As could be expected, selection for AUG is the strongest in slow evolving, highly expressed genes. We also obtained evidence that sub-optimality of the start codon potentially could be compensated by increased strength of the Shine-Dalgarno signal or by substitutions in positions −1 to −3 that, presumably, also enhance translation.

A somewhat unexpected result of the present analysis is the finding that selection affecting start codons is significantly weaker than that on the corresponding codon switches in coding regions that lead to amino acid replacements. The replacements involved are all conservative (switches between bulky, hydrophobic amino acids) and mildly deleterious at worst. Nevertheless, the present results indicate that selection against these amino acid changes is, on average, measurably stronger than selection against replacement of AUG with a sub-optimal start codon. Such weak selection on translation starts appears surprising given than AUG accounts for about 90% of all translation starts and implies that there is a sizable neutral range of translation initiation rates.

## Electronic supplementary material

Supplementary material
