# Supplementary material for: Selection on start codons in prokaryotes and potential compensatory nucleotide substitutions
Source: Sci Rep. 2017 Sep 29;7:12422. doi: 10.1038/s41598-017-12619-6 (PMC5622118; doi:10.1038/s41598-017-12619-6)
Supplement: Supplementary file 1 — Supplementary material [file 41598_2017_12619_MOESM1_ESM.pdf]

Selection on start codons in prokaryotes and potential compensatory nucleotide substitutions

Frida Belinky[1], Igor B. Rogozin[1], Eugene V. Koonin[1*]

[1]National Center for Biotechnology Information, National Library of Medicine, National Institutes of Health, Bethesda, Maryland, USA

[*]**To whom correspondence should be addressed. Email:** koonin@ncbi.nlm.nih.gov

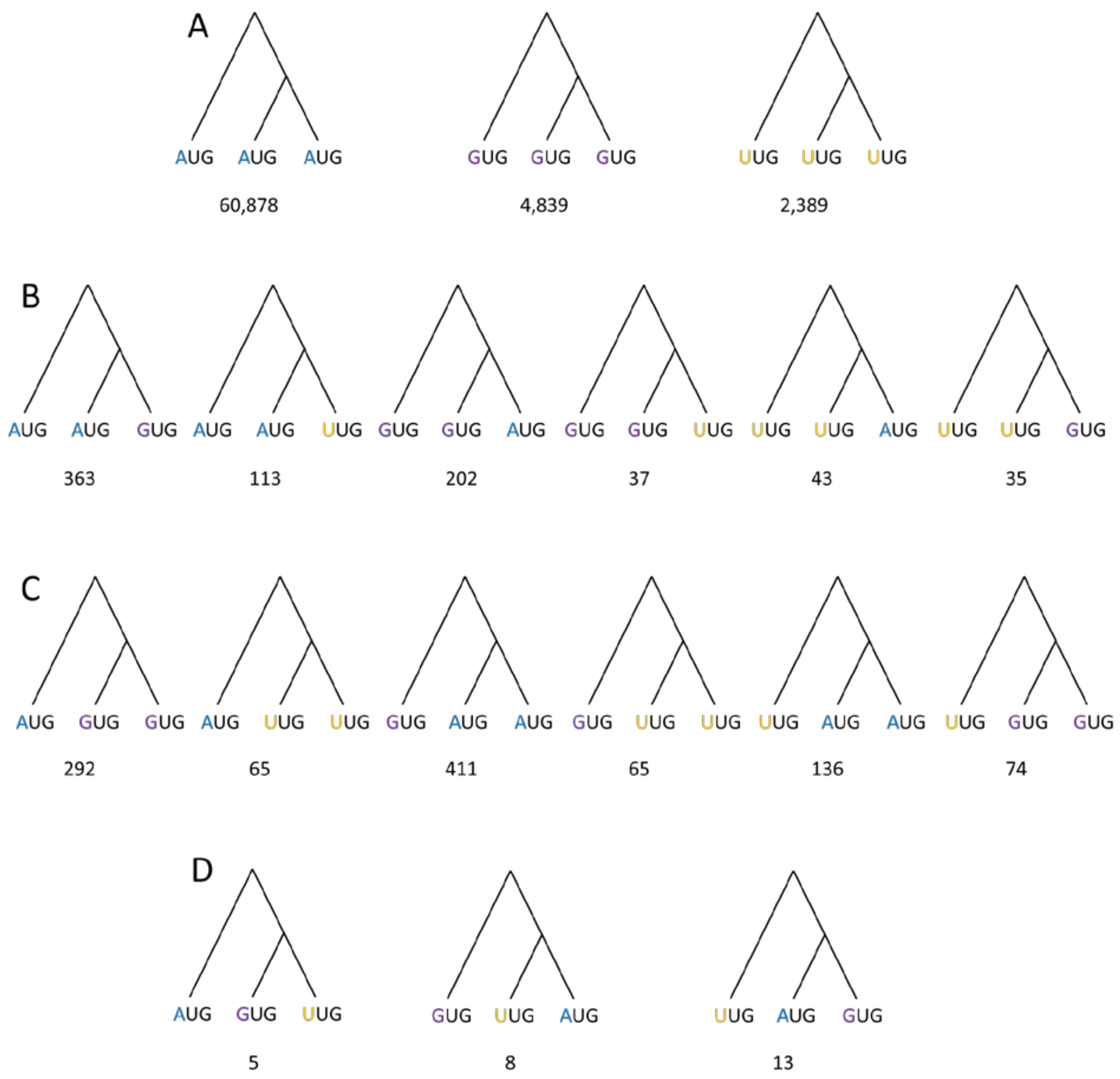

Figure S1. Configurations and gene counts for all start codon positions used for analyses of switch frequencies. Only configurations **A** and **B** were used to calculate switch frequencies limiting substitutions to the short external branch, under the parsimony assumption. **A** Identical start codon positions in all 3 related genomes. **B** configuration where one of the ingroup species has an identical start codon to the outgroup species. **C** Configurations where two ingroup species have an identical start codon and the outgroup species has a different one. **D** Configurations where all genomes have different start codons.

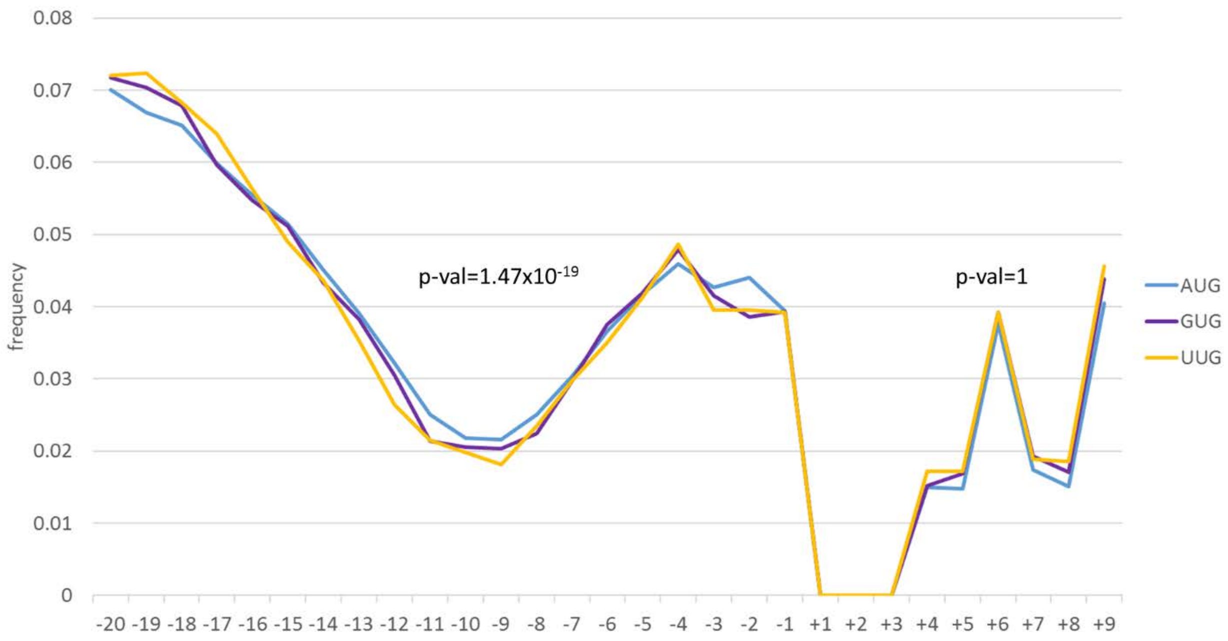

Figure S2. Cumulative substitution frequencies in 29 base pair windows. Based on pairwise comparison of closely related bacterial species with no more than 10 substitutions.

Position	A	B	C	D	E	F
+9						
+8						
+7						
+6			X			
+5						
+4						
+3					X	
+2						
+1	X	X	X			
-1	X			X		
-2						
-3						
-4				X		X
-5						X
-6						
-7						
-8						
-9		X			X	
-10						
-11						
-12						
-13						
-14						
-15						
-16						
-17						
-18						
-19						
-20						

	Substitutions at +1 and another at	Substitution at any but +1 and another at
-1, -2, -3	Example A	Example D
-7, -8, -9, -10	Example B	Example E
Other positions	Example C	Example F

Figure S3.

Examples of substitution pairs (A – F) across ATGCs and genes.

Each example adds to the count of one of the table's entries.

All substitution pair counts are summed up in Table 2.

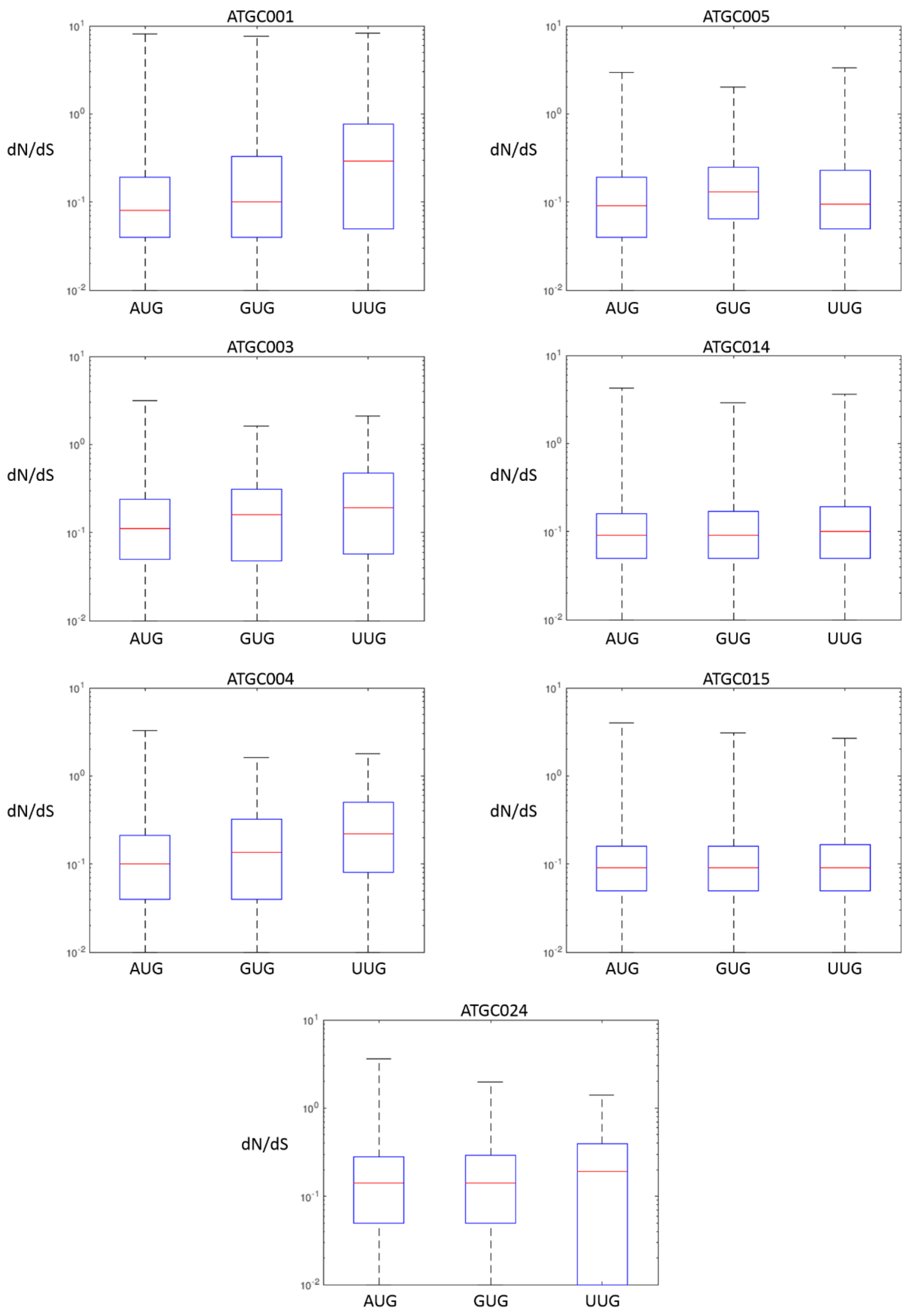

Figure S4 (part I)

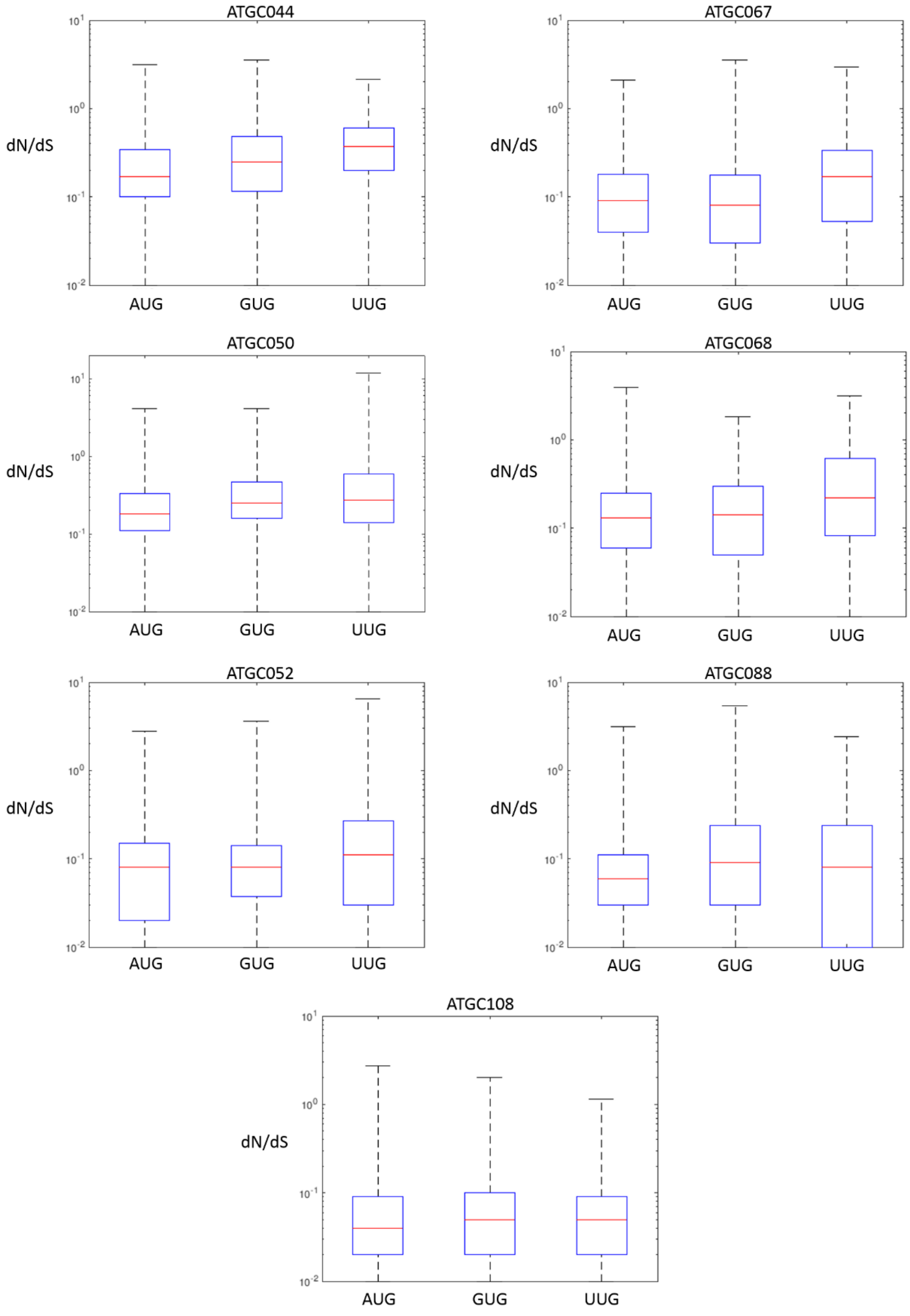

Figure S4 (part II)

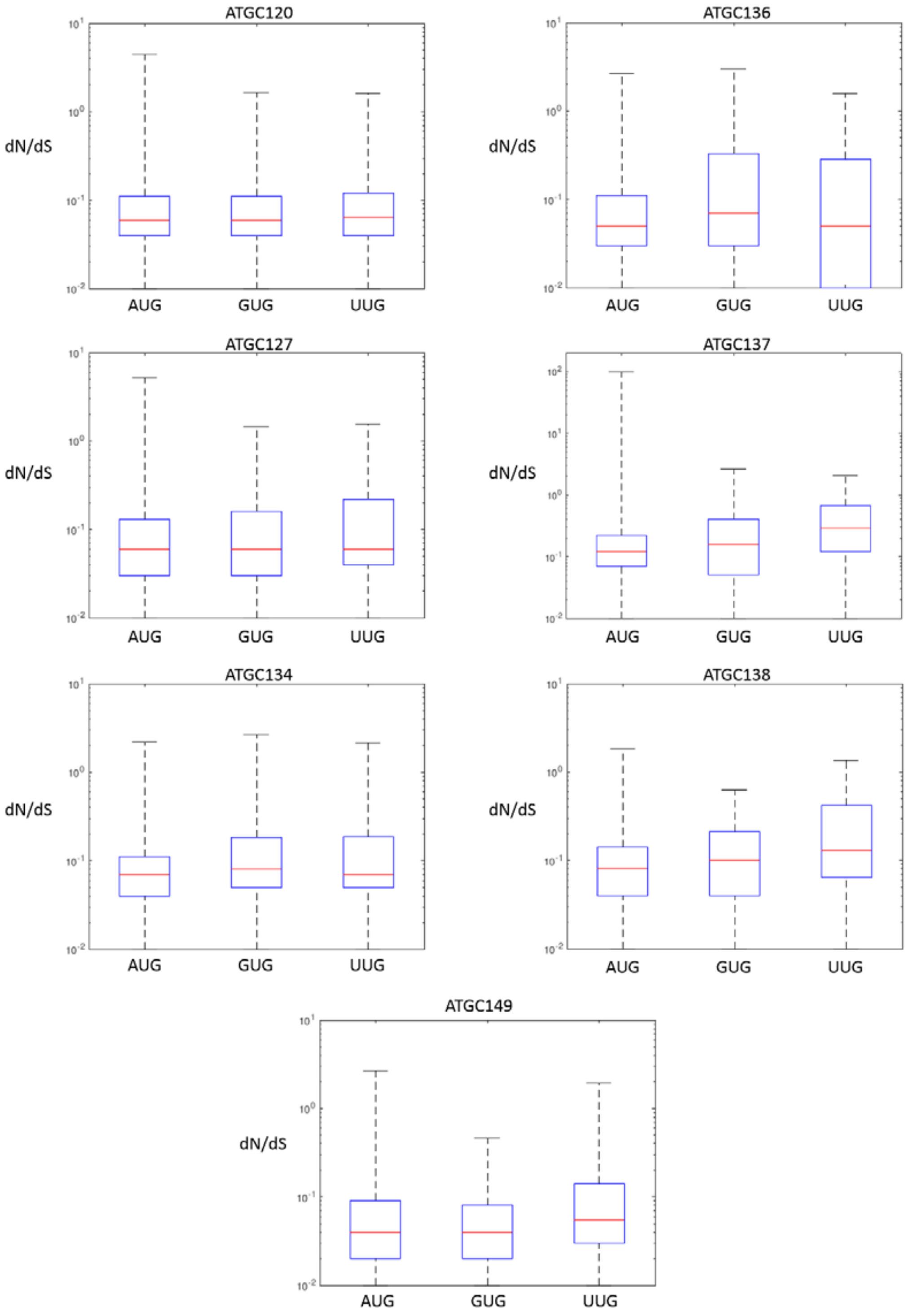

Figure S4. (part III). Evolutionary rates of genes starting with AUG, GUG and UUG in 21 ATGC groups with more than 12 species and more than 1,000 genes. The lower bound of all dN/dS values in all panels is zero. To enable presentation in log scale the lower bound was shifted to a finite value that was arbitrarily set to 0.01.

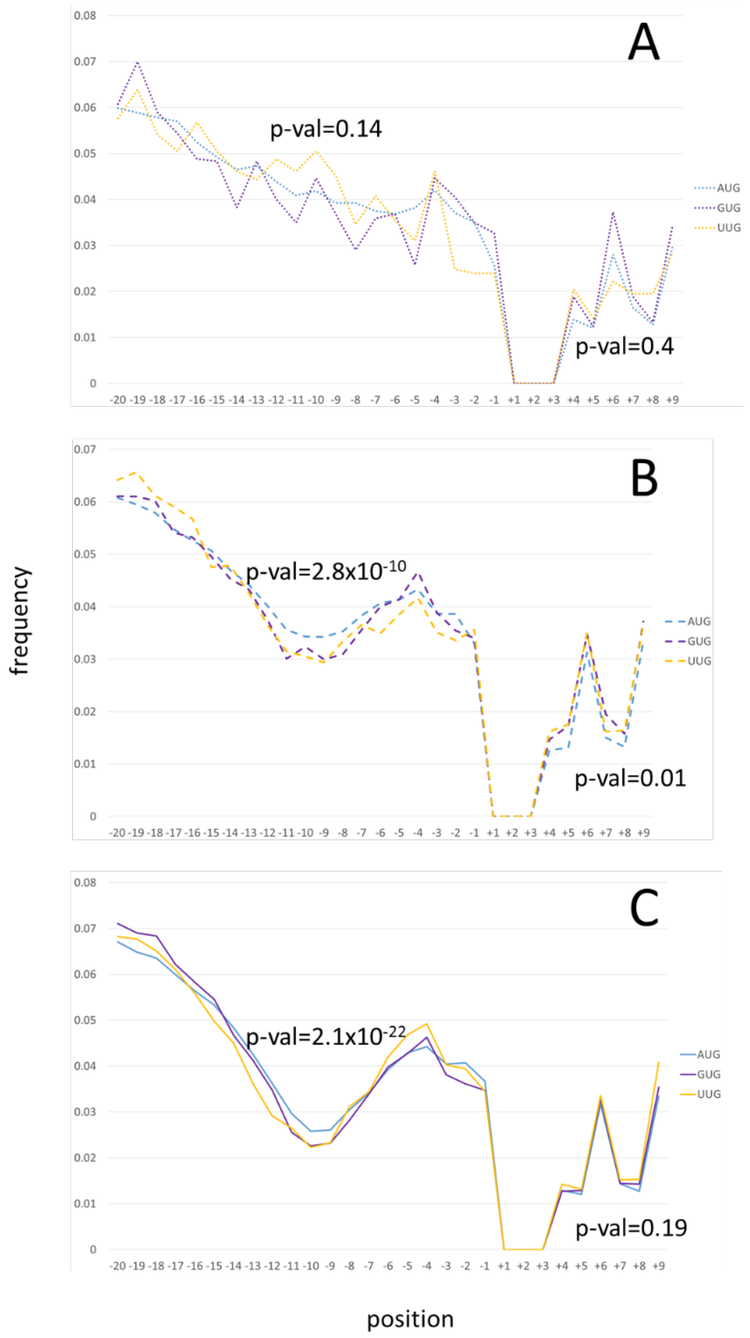

Figure S5. Cumulative substitution frequency grouped for different start codons and different strengths of Shine-Dalgarno. (A), no Shine-Dalgarno signal; (B), weak Shine-Dalgarno; (C), strong Shine-Dalgarno.

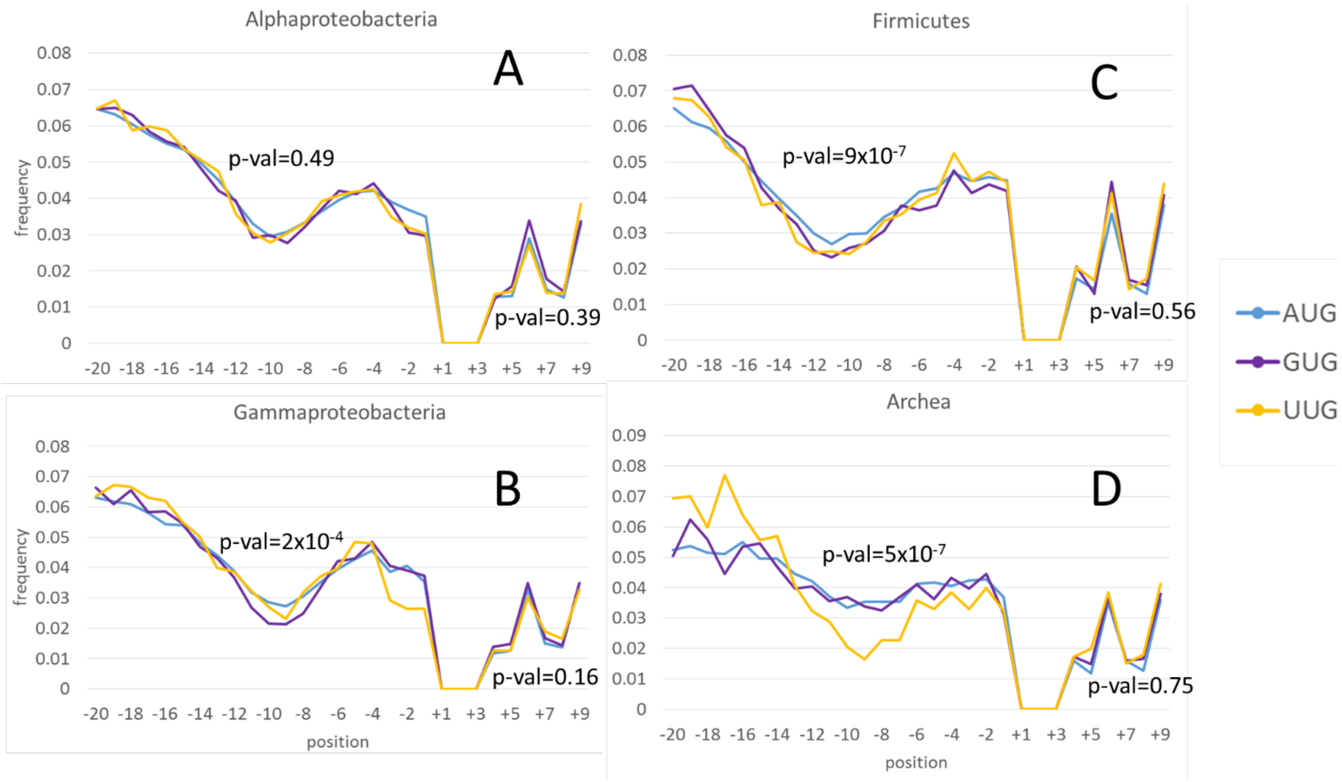

Figure S6. Cumulative substitution frequency grouped for different start codons in Alphaprotebacteria (A), Gammaproteobacteria (B), Firmicutes (C), and Archaea (D).

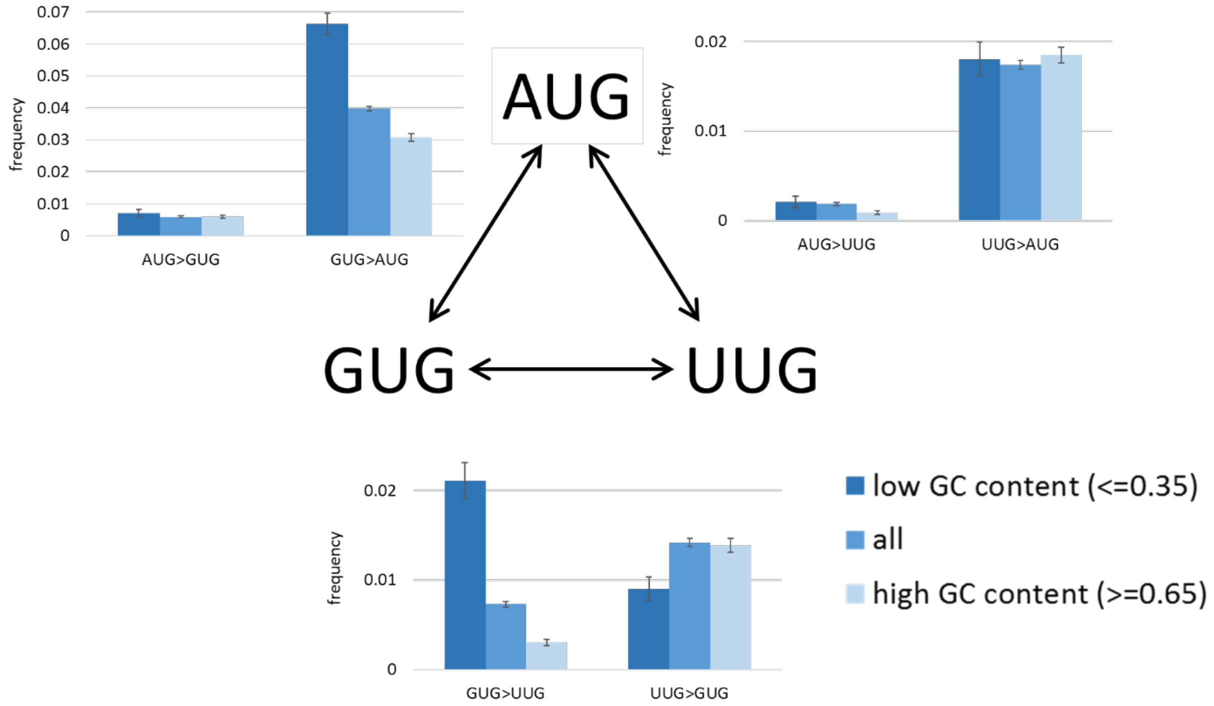

Figure S7. Switches between start-codons in 36 triples of prokaryotic genomes, compared between genes with low GC content (GC <=0.35) and genes with high GC content (GC >=0.65). The ‘all’ group includes additional taxa with 0.35<GC<0.65 and is therefore not always between the low and high GC groups.

Table S1. Counts and frequencies of start codon switches and ancestral start codon states after removal of genes with a conserved upstream start codon in the 60 upstream bases (denoted ‘no upstream start’), compared to all genes included in the analysis (denoted ‘all’) and compared to counts and frequencies after removal of genes with conserved start codons in the 60 downstream bases (denoted ‘no downstream start’).

	no upstream start		all		no downstream start	
	count	frequency	count	frequency	count	frequency
UUG>GUG	32	0.014337	35	0.014182	21	0.014103
GUG>UUG	34	0.007055	37	0.007283	27	0.008362
UUG>AUG	41	0.018369	43	0.017423	36	0.024177
AUG>UUG	108	0.001888	113	0.001842	87	0.002495
GUG>AUG	196	0.040672	202	0.039764	143	0.044286
AUG>GUG	353	0.006171	363	0.005916	258	0.007398
ancestral AUG	57200	0.890259	61358	0.890459	34873	0.880832
ancestral GUG	4819	0.075003	5080	0.073724	3229	0.081559
ancestral UUG	2232	0.034739	2468	0.035817	1489	0.03761

Table S2. Maximum Likelihood marginal probabilities for ancestral states of the different scenarios used for the switch frequency analyses of start codons. ‘A’ denotes AUG, ‘G’ denotes GUG and ‘T’ denotes UUG.

Outgroup	ingroup1	ingroup2	Parsimony reconstructed ancestral state	FastML marginal probabilities				ML support for parsimony ancestral site
				A	C	G	T	
A	A	G	A	0.942	0.0002	0.057	0.00036	0.942
A	A	U	A	0.958	0.0005	0.004	0.03728	0.958
A	U	A	A	0.962	0.0005	0.004	0.03388	0.962
A	G	A	A	0.947	0.0002	0.052	0.00035	0.947
G	G	A	G	0.0101	0	0.9895	0.00041	0.9895
G	A	G	G	0.009	0	0.9905	0.00039	0.9905
G	G	U	G	0.0001	0	0.966	0.03391	0.966
G	U	G	G	0.0001	0	0.969	0.03087	0.969
U	U	A	U	0.0028	0	0.001	0.996	0.996
U	A	U	U	0.0025	0	0.001	0.996	0.996
U	U	G	U	0	0	0.016	0.984	0.984
U	G	U	U	0	0	0.014	0.986	0.986

The marginal probabilities were estimated using FastML with the GTR substitution model.

Table S3. Original and sampled switch counts and frequencies for the 3 control groups in Fig. 1 and Table 1. Positions were sampled so the ancestral state frequencies would match those of the start codon ancestral frequencies. Although the ancestral states are varied in the sampled data compared to the original data, the switch frequencies remain the same.

4 fold degenerate site substitutions followed by UG	original count	original frequency	sampled mean count	sampled mean frequency	sample standard error of count**
AUG>UUG	601	0.04254	601	0.04254	0
AUG>GUG	1564	0.11071	1564	0.11071	0
GUG>UUG	649	0.01947	24.479	0.01931	0.15218
UUG>GUG	573	0.03140	18.458	0.03160	0.14105
UUG>AUG	628	0.03441	20.276	0.03472	0.14450
GUG>AUG	2807	0.08421	106.304	0.08387	0.32523
Ancestral AUG	14127	0.21498	14127	0.88412	0
Ancestral UUG	18250	0.27772	584.061	0.03655	00.75868
Ancestral GUG	33335	0.50729	1267.514	0.07933	1.092198
Coding substitutions	original count	original frequency	sampled mean count	sampled mean frequency	sample standard error of count*
AUG>UUG	1790	0.002708	1790	0.002708	0
AUG>GUG	2623	0.003968	2623	0.003968	0
GUG>UUG	1106	0.001508	83.2026	0.001512	5.03728
UUG>GUG	771	0.003794	101.5295	0.003814	0.29
UUG>AUG	948	0.004666	123.9428	0.004656	0.3294
GUG>AUG	3343	0.004559	250.6148	0.004556	0.4773
Ancestral AUG	660975	0.413752	660975	0.890083	0
Ancestral UUG	203189	0.127191	26617.5787	0.035844	5.0373
Ancestral GUG	733352	0.459058	55006.0762	0.074072	7.4338
Non-coding substitutions	original count	original frequency	sampled mean count	sampled mean frequency	sample standard error of count**
AUG>UUG	783	0.007855	783	0.007855	0
AUG>GUG	2273	0.022804	2273	0.022804	0
GUG>UUG	553	0.006505	53.6	0.006503	0.222
UUG>GUG	659	0.005612	22.54	0.005641	0.1486
UUG>AUG	722	0.006148	24.913	0.006235	0.1559
GUG>AUG	2370	0.027879	229.857	0.027887	0.4496
Ancestral AUG	99677	0.329936	99677	0.890648	0
Ancestral UUG	117423	0.388676	3995.685	0.035703	2.0006
Ancestral GUG	85010	0.281388	8242.515	0.07365	2.7694

* All AUG cases were included in all the samples, thus the standard error for all switches from AUG is zero, positions containing GUG and UUG were sampled to match the start codon frequencies.

Table S4. Comparison of the *dN/dS* values for genes starting with AUG, GUG and UUG in 21 ATGC groups. Wilcoxon rank sum test was performed on the dN/dS values of genes starting with different start codons in each group. Fisher's exact test was performed on the number of genes starting with each codon and having higher or lower dN/dS compared to the overall median in each ATGC group. The ATGC groups included are the ones that have more than 12 species and more than 1,000 total genes.

ATGC group	# species	median dN/dS of genes starting with			Wilcoxon rank sum test			Fisher's exact test			# genes starting with		
		AUG	GUG	UUG	AUG vs. GUG	AUG vs. UUG	GUG vs. UUG	AUG vs. GUG	AUG vs. UUG	GUG vs. UUG	AUG	GUG	UUG
ATGC001	109	0.08	0.1	0.29	2.7E-05	7.4E-20	8.8E-08	3.1E-03	1.6E-06	0.02	6119	745	328
ATGC003	22	0.11	0.16	0.19	0.12	2.1E-03	0.32	0.05	0.06	0.88	1546	77	101
ATGC004	22	0.1	0.14	0.22	0.03	6.2E-09	0.06	0.05	4.5E-05	0.26	1332	80	108
ATGC005	16	0.09	0.13	0.1	0.05	0.36	0.41	0.01	0.76	0.13	1210	48	50
ATGC014	31	0.09	0.09	0.1	0.07	1.4E-05	0.07	0.43	5.4E-04	0.06	5523	623	654
ATGC015	24	0.09	0.09	0.09	0.82	0.52	0.79	0.50	0.93	0.70	3228	468	572
ATGC024	32	0.14	0.14	0.19	0.88	0.31	0.33	0.55	0.29	0.19	1376	754	100
ATGC044	40	0.17	0.25	0.37	4.6E-03	5.9E-12	0.03	1.1E-03	5.4E-14	0.01	1231	104	154
ATGC050	51	0.18	0.25	0.27	1.7E-04	2.0E-08	0.54	1.9E-03	4.3E-06	0.91	1265	121	239
ATGC052	42	0.08	0.08	0.11	0.50	6.5-05	0.02	0.66	0.01	0.22	1859	137	199
ATGC067	18	0.09	0.08	0.17	0.20	2.0E-03	1.7E-03	0.10	0.06	0.01	1208	351	75
ATGC068	13	0.13	0.14	0.22	0.15	2.0E-04	9.2E-03	0.62	0.04	0.09	1100	346	75
ATGC088	13	0.06	0.09	0.08	3.7E-08	1.6E-03	0.47	0.16	0.16	0.75	4031	496	250
ATGC108	31	0.04	0.05	0.05	0.23	0.35	0.75	0.40	0.40	1	1946	206	258
ATGC120	14	0.06	0.06	0.07	0.86	0.30	0.45	0.75	0.52	0.74	3772	343	156
ATGC127	19	0.06	0.06	0.06	0.72	0.41	0.56	0.47	0.90	0.89	2825	239	78
ATGC134	13	0.07	0.08	0.07	1.2E-07	0.02	0.65	1.7E-03	0.07	1	3164	433	123
ATGC136	19	0.05	0.07	0.05	0.16	0.57	0.28	0.43	0.46	0.26	1190	93	75
ATGC137	18	0.12	0.16	0.29	0.04	1.0E-09	0.03	0.13	2.4E-06	0.03	1720	80	90
ATGC138	18	0.08	0.1	0.13	0.09	1.2E-03	0.19	0.10	2.6E-03	0.37	1240	45	47
ATGC149	14	0.04	0.04	0.06	0.04	0.09	0.01	0.02	0.89	0.15	2972	164	74

Table S5. Counts and ratios for combinations of start codon and start codon switches with Shine-Dalgarno strength and Shine-Dalgarno strength switches. Fisher's exact test was used to determine significance.

	strong	weak	ratio	p-val
AUG	20597	12340	0.599116	5.53E-05
GUG	1631	818	0.501533	

	strong	weak	ratio	p-val
AUG	20597	12340	0.599116	1.64E-10
UUG	959	392	0.408759	

	strong>weak	weak>strong	ratio	p-val
AUG	544	385	0.707721	0.1743
GUG	29	30	1.034483	

	strong>weak	weak>strong	ratio	p-val
AUG	544	385	0.707721	0.6126
UUG	20	17	0.85	

	strong	weak	ratio	p-val
AUG>GUG	75	46	0.613333	0.4071
GUG>AUG	41	18	0.439024	

	strong	weak	ratio	p-val
AUG>UUG	23	19	0.826087	1
UUG>AUG	10	8	0.8	

	strong>weak	weak>strong	ratio	p-val
AUG>GUG	32	10	0.3125	0.1022
GUG>AUG	4	5	1.25	

	strong>weak	weak>strong	ratio	p-val
AUG>UUG	17	5	0.294118	1
UUG>AUG	11	3	0.272727	

Table S6. Counts of major start codons in 36 genome triplets included in the switch analyses.

ATGC	outgroup	ingroup1	ingroup2	outgroup	ingroup1	ingroup2	outgroup	ingroup1	ingroup2
	AUG			GUG			UUG		
ATGC001	1982	1982	1980	121	121	124	20	20	19
ATGC003	1090	1087	1093	25	31	25	20	17	17
ATGC008	847	857	858	45	43	43	43	35	34
ATGC014	1847	1848	1854	165	161	156	130	133	132
ATGC015	2001	1998	2005	193	194	185	250	251	254
ATGC035	116	117	117	3	2	2	1	1	1
ATGC044	249	250	248	18	17	19	3	2	3
ATGC050	829	830	831	46	49	48	78	74	74
ATGC071	3424	3423	3421	262	265	261	58	56	62
ATGC088	2409	2413	2403	129	128	135	46	43	46
ATGC089	3314	3312	3316	185	185	181	74	76	76
ATGC097	1833	1834	1833	143	138	142	34	38	35
ATGC100	2458	2452	2449	177	183	184	34	34	36
ATGC104	791	800	797	92	84	85	21	20	22
ATGC108	1835	1845	1835	165	163	173	202	194	194
ATGC111	2060	2064	2062	110	111	113	44	39	39
ATGC123	3369	3360	3366	217	220	219	119	125	120
ATGC125	2862	2878	2876	180	167	168	92	89	89
ATGC134	1522	1535	1535	143	135	138	33	28	25
ATGC135	2492	2488	2486	223	222	224	37	42	42
ATGC137	802	799	801	23	25	25	8	9	7
ATGC144	551	552	552	41	39	40	79	81	80
ATGC147	1222	1223	1223	52	53	53	104	101	102
ATGC149	2023	2019	2021	109	111	110	21	23	22
ATGC165	1872	1881	1879	138	133	135	58	54	54
ATGC171	1087	1088	1097	132	132	128	130	129	124
ATGC177	1113	1114	1114	114	113	114	138	138	137
ATGC181	1167	1175	1165	135	141	144	205	193	200
ATGC188	1866	1866	1866	79	76	80	32	35	31
ATGC189	2994	3002	3001	158	164	161	52	40	44
ATGC199	2302	2315	2310	228	218	217	238	235	239
ATGC201	860	861	857	54	54	56	19	18	20
ATGC210	739	736	735	69	74	74	70	68	69
ATGC213	2325	2325	2326	318	319	315	94	93	96
ATGC234	1077	1083	1089	534	526	524	23	24	21
ATGC252	2390	2395	2396	738	735	734	81	79	79
